# A curative-intent endoscopic surgery for postradiation nasopharyngeal necrosis in patients with nasopharyngeal carcinoma

**DOI:** 10.1186/s40880-018-0338-4

**Published:** 2018-12-22

**Authors:** Xiong Zou, Shun-Lan Wang, You-Ping Liu, Yan-Ling Liu, Ru-Hai Zou, Yi-Nuan Zhang, Rui You, Qi Yang, Yu-Long Xie, Mei Lin, Pei-Yu Huang, Rou Jiang, Meng-Xia Zhang, Chao-Nan Qian, Hai-Qiang Mai, Ling Guo, Ming-Huang Hong, Ming-Yuan Chen

**Affiliations:** 1 State Key Laboratory of Oncology in South China, Collaborative Innovation Center for Cancer Medicine, Sun Yat-sen University Cancer Center, Guangzhou, 510060 Guangdong P.R. China; 2 Department of Nasopharyngeal Carcinoma, Sun Yat-sen University Cancer Center, 651 Dongfeng Road East, Guangzhou, 510060 Guangdong P.R. China; 3 Guangdong Key Laboratory of Nasopharyngeal Carcinoma Diagnosis and Therapy, Sun Yat-sen University Cancer Center, Guangzhou, 510060 Guangdong P.R. China; 4 Department of Otorhinolaryngology, First Hospital Affiliated of Guangzhou University of Traditional Chinese Medicine, Guangzhou, 510405 Guangdong P.R. China; 5 Department of Ultrasound, Sun Yat-sen University Cancer Center, Guangzhou, 510060 Guangdong P.R. China

**Keywords:** Nasopharyngeal carcinoma, Postradiation, Necrosis, Endoscopy, Necrectomy, Flap, Reconstruction, Re-epithelialization

## Abstract

**Background:**

Postradiation nasopharyngeal necrosis (PRNN) is a severe complication after radiotherapy in patients with nasopharyngeal carcinoma (NPC), which can severely affect the quality of life and threaten the patient’s life. Only 13.4%–28.6% of patients can be cured by traditional repeated endoscopic debridement. Here, we introduced an innovative curative-intent endoscopic surgery for PRNN patients and evaluated its clinical efficacy.

**Methods:**

Clinical data of 72 PRNN patients who underwent radical endoscopic necrectomy, followed by reconstruction using a posterior pedicle nasal septum and floor mucoperiosteum flap were analyzed to determine the efficacy of this surgery. The endpoints were complete re-epithelialization of the nasopharyngeal defect, relief of headache, and overall survival (OS).

**Results:**

All surgeries were successfully performed without any severe postoperative complications or death. The median value of numeric rating scales of pain decreased from 8 before surgery to 0 after surgery (*P *< 0.001). Fifty-one patients (70.8%) achieved complete re-epithelialization of the nasopharyngeal defect. The number of cycles of radiotherapy (odds ratio [OR], 7.254; 95% confidence interval [CI] 1.035–50.821; *P *= 0.046), postoperative pathological result (OR, 34.087; 95% CI 3.168–366.746; *P *= 0.004), and survival status of flap (OR, 261.179; 95% CI 17.176–3971.599; *P *< 0.001) were independent risk factors of re-epithelialization of the nasopharyngeal defects. Postoperative pathological result (hazard ratio [HR], 5.018; 95% CI 1.970–12.782; *P *= 0.001) was an independent prognostic factor for OS. The 2-year OS rate of the entire cohort was 77.9%.

**Conclusion:**

Curative-intent endoscopic necrectomy followed by construction using the posterior pedicle nasal septum and floor mucoperiosteum flap is a novel, safe, and effective treatment of PRNN in patients with NPC.

## Background

Nasopharyngeal carcinoma (NPC) is a common malignancy in Southeast Asia, particularly in the Guangdong province of China [1–3]. Radiotherapy is a fundamental treatment of NPC, with an observed 5-year overall survival (OS) rate of 68.2%–87.4% for patients with non-metastatic NPC [4–7]. However, radiotherapy may cause acute and chronic adverse effects on the normal tissues and bones surrounding the nasopharynx. As a severe adverse event after radiotherapy, postradiation nasopharyngeal necrosis (PRNN) can be observed in NPC patients after receiving high-dose irradiation [8–10]. It is commonly accompanied by a severe headache, foul odor, and epistaxis, which severely affect the patients’ quality of life [8, 9, 11]. Moreover, PRNN has been observed to contribute to 41.8%–42.9% of NPC patients’ death and to increase the risk of death by 69.2%–72.7% when the internal carotid artery (ICA) is exposed in the PRNN lesions [11, 12]. As such, ICA exposure has been found as an independent prognostic factor for patients with PRNN [8, 9, 11, 12]. Although repeated endoscopic debridement was reported as an effective traditional treatment of PRNN, only 13.4%–28.6% of these patients could be cured [11, 12], reflecting the possibility that the necrotic tissue could not be completely removed with or without efficient re-epithelialization of the nasopharyngeal defect.

Recently, we developed a new reconstruction technique using the posterior pedicle nasal septum and floor mucoperiosteum flap based on the posterior nasal septal artery to treat the nasopharyngeal defect, which has shown to promote wound healing and successfully resurface fresh, clean wounds after endoscopic nasopharyngectomy for patients with locally recurrent NPC [13]. However, whether this technique would be effective in treating old and infected PRNN wounds remains unclear. Thus, in this present study, we aimed to investigate and evaluate the clinical efficacy of a novel curative-intent endoscopic surgery comprising of radical endoscopic necrectomy and reconstruction of the nasopharyngeal defect using the nasal septum and floor mucoperiosteum flap to treat the PRNN lesions of NPC patients.

## Patients and methods

### Patient selection

From July 2010 to January 2017, NPC patients who had radical endoscopic necrectomy followed by reconstruction using a nasal septum and floor mucoperiosteum flap at the Sun Yat-sen University Cancer Center were selected for enrollment. This study was approved by the ethical committee of Sun Yat-sen University Cancer Center. Written informed consents were obtained from all patients.

The selection criteria were as follows: (1) a definitive history of radiotherapy, (2) diagnosed with PRNN based on clinical symptoms, imaging examinations, and preoperative biopsy, (3) their PRNN lesion was limited to a resectable region (i.e., the ICA was located at the outside margin of the lesion, the upper bound of the lesion consisted of the roof of the sphenoid sinus and ethmoid sinus, the posterior boundary of the lesion did not exceed the clivus, and the lower edge of axis was located at the lower bound of the lesion) so that the necrotic tissue could be completely removed with curative-intent.

The exclusion criteria for this study were as follows: (1) broad osteoradionecrosis of the skull base exceeding out of the resectable region (e.g. the lower edge of the axis) which would render the removal with curative-intent difficult and risky, (2) pathologically confirmed local recurrence with or without distant metastases before surgery, and (3) physical conditions unsuitable for surgery, such as heart failure, severe cardiac tamponade, unstable hemodynamics, severe nervous system abnormalities, clinically apparent malperfusion including lower limb, cerebral, coronary and renal malperfusion, and visceral ischemia or severe hepatic and renal abnormalities.

### Surgery

Prior to surgery, a bacterial cultivation for purulent nasopharyngeal secretions and drug sensitivity assays were performed to identify sensitive antibiotics to be used postoperatively. For patients with ICA exposure in the PRNN lesions, a balloon occlusion test (BOT) was conducted to determine if the ICA could be safely occluded without causing cerebral ischemia [14, 15], and endovascular coiling embolization was required for some patients who suffered from vascular wall trauma or pseudoaneurysm, in order to avoid sudden fatal nasopharyngeal hemorrhage during surgery.

**Fig. 1:**
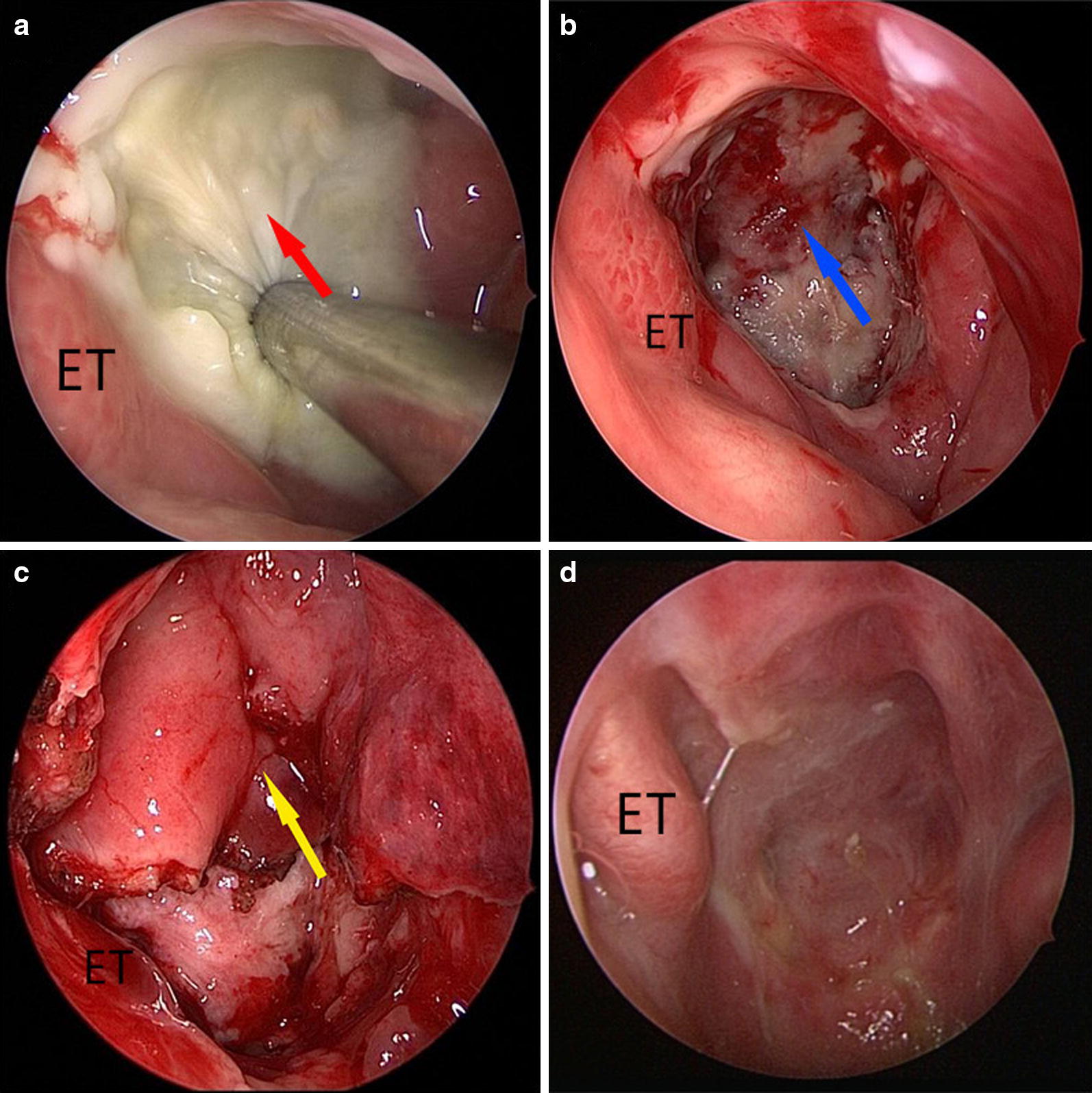
The radical endoscopic necrectomy followed by reconstruction using a posterior pedicle nasal septum and floor mucoperiosteum flap to resurface the nasopharyngeal defects in a 62-year-old man with post-radiation nasopharyngeal necrosis (PRNN). **a**–**d** display the anterior views of the nasal and nasopharyngeal cavity. **a** After full exposure of the nasopharyngeal cavity, the PRNN (indicated by the red arrow) in the roof of the posterior wall of the nasopharyngeal cavity was observed after removing purulent nasopharyngeal secretions using a steel suction apparatus. **b** After radical endoscopic necrectomy, the skull base (indicated by the blue arrow) and normal tissues were exposed. **c** In the ipsilateral nasal cavity, the flap (indicated by the yellow arrow) re-covered the nasopharyngeal defect. **d** Eight weeks after surgery, the NSFF completely relined the nasopharyngeal mucosa. *ET* eustachian tube, *NSFF* posterior pedicle nasal septum, and floor mucoperiosteum flap

**Fig. 2:**
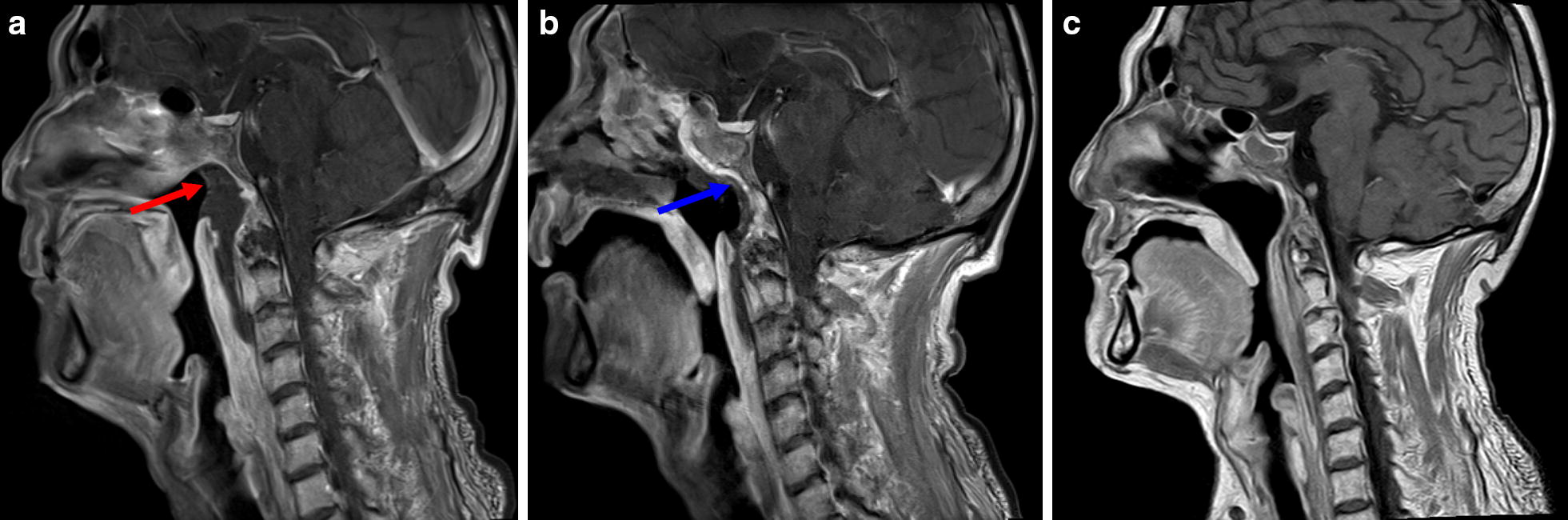
Contrast-enhanced sagittal magnetic resonance imaging (MRI) of the head and neck of a patient before and after surgery. **a** PRNN (indicated by the red arrow) was located in the roof of the posterior wall of the nasopharyngeal cavity and oral pharynx as shown on the preoperative MRI. **b** The flap (indicated by the blue arrow) re-surfaced the defect after radical endoscopic necrectomy, with good blood supply at 2 weeks after surgery. **c** The flap completely relined the nasopharyngeal mucosa at 8 weeks after surgery

Radical endoscopic necrectomy was performed under general anesthesia using the endoscopic endonasal approach. The nasopharyngeal necrotic soft tissue (Figs. 1a, 2a) was completely removed using the XPS 3000 powered ENT system (Medtronic, Minneapolis, MN, USA) until a slight bleeding was observed (Fig. 1b), thereby exposing the underlying normal fresh tissue. Electrocoagulation was then performed to remove tough connective tissues surrounding the fresh tissues using a long head electrotome (Covidien, Saint Louis, MO, USA). If the skull base bone and anterior arch of atlas were observed to be necrotized, they were removed using a high-speed electric micro-drill (Stryker, Kalamazoo, MI, USA). Surgical specimens from the lateral and basal surgical margins, any tissue suspected to be a recurrent tumor, and the necrotic tissues removed were sent for pathological examination. One side flap, typically the ipsilateral side, was harvested after complete radical endoscopic necrectomy, as reported in our previous study [13]. According to the area of the nasopharyngeal defect, except for a narrow posterior pedicle above the nasal lumen, the flap which contained the posterior nasal septal artery was partially or completely separated and gently rotated backward to cover the defect (Figs. 1c, 2b). If the one-side flap was not large enough to cover the entire defect, the flap on the opposite side of the nasal septum was also simultaneously harvested with the inferior turbinate mucoperiosteum being separated to cover the oropharyngeal defect, especially when the necrotic anterior arch of the atlas was removed. Subsequently, an absorbable gelatin sponge was gently placed into the nasopharyngeal cavity to support the flap. A Rapid Rhino Nasal Pac with Gel Knit (Applied Therapeutics, Tampa, FL, USA) was used to hold up the absorbable gelatin sponge from the nasal cavity to avoid it falling into the oropharyngeal cavity. If the flap necrotized after being harvested, the flap on the opposite side of nasal septum was harvested as a remedial measure. If the flap with two bared sides of the nasal septum were harvested, the nasal septum bone would be removed.

Antibiotic therapy based on the result of bacterial cultivation before the surgery was administered for 5–7 days after surgery. Magnetic resonance imaging (MRI) of the nasopharynx was performed within 2 weeks after the operation to evaluate the flap blood supply (Fig. 2b). When signs of poor blood supply were observed on the MRI, balloon outgassing was performed to relieve the flap compression, and additional treatment with vasoactive drugs was prescribed. After removing the nasal packing on the 7th postoperative day, the patients were prescribed regional perfusion with recombinant human epidermal growth factor derivative liquid (Watsin Genetech Ltd, Shenzhen, Guangdong, P.R. China) to keep the wound humid, and endoscopic nasal cleanup was performed every 2–4 weeks until the flap was completely epithelialized and the entire defect was relined (Figs. 1d, 2c).

### Evaluation of treatment efficacy

Locoregional recurrence in this study was defined as tumor recurrence identified in the PRNN lesion with positive pathology at the time of surgery or if the PRNN lesion had negative pathology at the time of surgery but the patient developed recurrence during follow-up examinations.

The primary endpoint of this study was the relining of the nasopharyngeal defect which was defined as the complete re-epithelialization of the nasopharyngeal cavity, upon nasal endoscopic examination, and the mucosa; upon MRI examination [8]. The observed changes in headache episodes and OS were defined as the secondary endpoints. The Numeric Rating Scales (NRS) of pain was used to evaluate headaches level before and within 3 months after surgery. The patients were requested to rate their level of headache on a scale from 0 to 10, with 0 being none and 10 being unbearable. OS was calculated from the date of PRNN surgery till the date of last follow-up (September 20, 2017) or death.

### Follow-up

All patients were followed-up to assess the surgical outcomes, disease status, and performance status every 3 months in the first 3 years after the operation, every 6 months in the fourth and fifth years, and annually thereafter. Follow-up examinations included complete physical examinations, chest X-ray, abdominal ultrasonography, MRI of the head and neck, and/or whole-body bone scan.

### Statistical analysis

For all cohorts, categorical data were described as numbers and percentages, and continuous data are presented as medians. Categorical data were analyzed using the Chi-square test or Chi-square test with continuity correction. Wilcoxon rank-sum test was conducted for ranked data. Univariate logistic regression was used to determine the associations between clinicopathological characteristics and complete re-epithelialization of the nasopharyngeal defect. Variables associated with severe necrosis (*P *< 0.1) in univariate logistic regression analysis were included in multivariate logistic regression models to adjust for confounding effects. Survival results were calculated using the Kaplan–Meier method, and their differences were compared using the log-rank test. Covariates significantly associated with prognosis detected in univariate analysis (*P *< 0.1) were included in a multivariate Cox proportional hazards model to identify independent prognostic factors. All analyses were performed using the SPSS software (version 16.0, SPSS, Chicago, IL, USA), and a two-tailed *P* value less than 0.05 was considered as statistically significant.

## Results

### Patient characteristics and surgical outcomes

Clinical records of 72 NPC patients, including 52 males and 20 females, with a median age of 51 years (range, 32–73 years) were selected. PRNN was diagnosed after one cycle of irradiation in 50 patients, with a median interval of 8 months (range, 1–240 months), and after two cycles of irradiation in 22 patients, with a median interval of 3.5 months (range, 0–103 months). PRNN was observed to occur within 3 months after radiotherapy (acute necrosis) in 17 patients and after 3 months (chronic necrosis) in 55 patients. Fifty-one patients presented with osteoradionecrosis, which included 5 patients who had combined necrosis of the anterior arch of the atlas. Fourteen patients presented with radiation-induced encephalopathy or temporal lobe necrosis with a median interval of 8 months (range, 2–150 months). Among the 25 patients whose ICA was exposed in their necrotic lesion, 24 had BOT intervention, and the remaining 1 patient rejected the BOT intervention. Among the 24 patients who underwent the BOT intervention, 14 of them underwent endovascular coiling embolization after the BOT.

Endoscopic necrectomy followed by reconstruction using the nasal septum and floor mucoperiosteum flap was successfully performed in all patients without any severe postoperative complications or death. The median surgical time was 110 min (range, 50–180 min) with little blood loss (median, 50 mL; range, 10–300 mL). Two patients had their flaps simultaneously harvested from both sides of the nasal septum in one operation. One patient underwent a repeated operation to harvest the flap on the opposite side of the nasal septum because the one-side flap necrotized 1 week after the surgery. Six patients had their inferior turbinate mucoperiosteum combined with the flap. Overall, the flap survived in 56 (77.8%) patients, and for the remaining 16 (22.2%) patients, their flaps became necrotic due to ischemia (Table 1). No clinical characteristic was significantly associated with the status of the flap (Table 2). Twenty patients were diagnosed with recurrent undifferentiated non-keratinizing carcinoma according to the findings of postoperative pathological assessments for deep surgical margins (Table 1).

**Table 1:** Associations between the clinical characteristics and the re-epithelialization of the nasopharyngeal defects for patients with postradiation nasopharyngeal necrosis who underwent radical necrectomy followed by reconstruction using posterior pedicle nasal septum and floor mucoperiosteum flap (NSFF)

Clinical characteristic	Total [cases (%)]	Re-epithelialization [cases (%)]		Univariate analysis		Multivariate analysis	
		Survived	Necrotized	*P* value	OR (95% CI)	*P* value	OR (95% CI)
Total	72	51 (70.8)	21 (29.2)				
Gender							
Female	20 (27.8)	11 (21.6)	9 (42.9)				
Male	52 (72.2)	40 (78.4)	12 (57.1)	0.072	2.727 (0.915–8.127)	0.778	1.328 (0.185–9.533)
Age (years)							
< 51	32 (44.4)	21 (41.2)	11 (52.4)				
≥ 51	40 (55.6)	30 (58.8)	10 (47.6)	0.386	0.636 (0.229–1.768)		
Cycles of radiotherapy							
One	50 (69.4)	40 (78.4)	10 (47.6)				
Two	22 (30.6)	11 (21.6)	11 (52.4)	0.012	4.000 (1.351–11.845)	0.046	7.254 (1.035–50.821)
Necrosis-free interval							
Acute necrosis	17 (23.6)	14 (27.5)	3 (14.3)				
Chronic necrosis	55 (76.4)	37 (72.5)	18 (85.7)	0.240	2.270 (0.578–8.919)		
Carotid artery exposure							
Yes	25 (34.7)	19 (37.3)	6 (28.6)				
No	47 (65.3)	32 (62.7)	15 (71.4)	0.483	0.674 (0.223–2.032)		
Osteoradionecrosis							
Yes	51 (70.8)	35 (68.6)	16 (76.2)				
No	21 (29.2)	16 (31.4)	5 (23.8)	0.522	1.463 (0.456–4.692)		
Postoperative pathological result							
Negative	52 (72.2)	42 (82.4)	10 (47.6)				
Positive	20 (27.8)	9 (17.6)	11 (52.4)	0.004	5.133 (1.677–15.714)	0.004	34.087 (3.168–366.746)
Status of the NSFF flap							
Survived	56 (77.8)	49 (96.1)	7 (33.3)				
Necrotized	16 (22.2)	2 (3.9)	14 (66.7)	< 0.001	49.000 (9.133–262.904)	< 0.001	261.179 (17.176–3971.599)

*OR* odds ratio, *CI* confidence interval, *NSFF* posterior pedicle nasal septum, and floor mucoperiosteum flap

**Table 2:** Associations between clinical characteristics and the status of posterior pedicle nasal septum and the floor mucoperiosteum flap

Characteristic	Status of the flap [cases (%)]		*P* value
	Survived	Necrotized	
Total	56	16	
Gender			0.193*
Female	13 (23.2)	7 (43.8)	
Male	43 (76.8)	9 (56.2)	
Age (years)			0.949
< 51	25 (44.6)	7 (43.8)	
≥ 51	31 (55.4)	9 (56.2)	
Cycles of radiotherapy			0.321*
One	41 (73.2)	9 (56.2)	
Two	15 (26.8)	7 (43.8)	
Necrosis-free interval			0.394*
Acute necrosis	15 (26.8)	2 (12.5)	
Chronic necrosis	41 (73.2)	14 (87.5)	
Carotid artery exposure			0.741
Yes	20 (35.7)	5 (31.2)	
No	36 (64.3)	11 (68.8)	
Osteoradionecrosis			0.177*
Yes	37 (66.1)	14 (87.5)	
No	19 (33.9)	2 (12.5)	
Postoperative pathological result			0.972*
Negative	41 (73.2)	11 (68.8)	
Positive	15 (26.8)	5 (31.2)	

* *P* values were calculated using the Chi square test with continuity correction

### Treatment efficacy

**Fig. 3:**
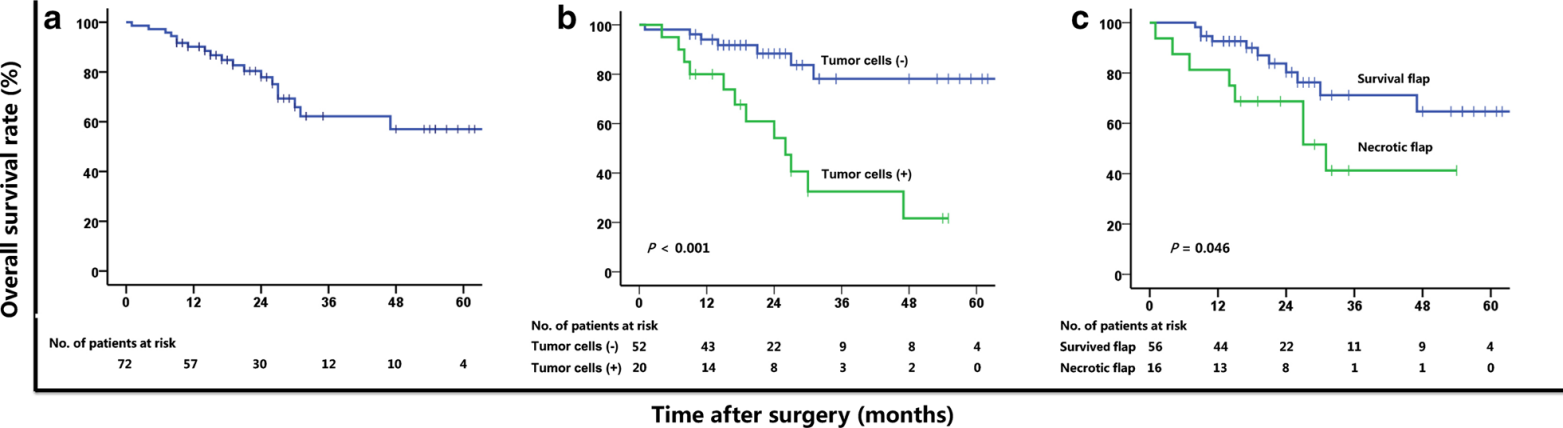
Kaplan–Meier curves illustrating the overall survival (OS) of **a** all patients, **b** patients stratified by their postoperative pathological result, and **c** patients stratified by the status of the flap. (*+*) positive, (*−*) negative, *Path* pathology

During the follow-up period (median, 20 months; range, 1.0–77.0 months), the nasopharyngeal defects were completely re-epithelialized in 51 (70.8%) patients, and the median time to re-epithelialization was 2.0 months (range, 1.1–3.3 months). The flaps did not show favorable re-epithelialization in the remaining 21 (29.2%) patients. Among the 50 patients who were receiving 1 course of radiotherapy, the nasopharyngeal defects were completely re-epithelialized in 40 (80.0%) patients, and the median time to re-epithelialization was 2.0 months (range, 1.3–26.0 months). Among the 22 patients who were receiving 2 courses of radiotherapy, the nasopharyngeal defects were completely re-epithelialized in 11 (50.0%) patients, and the median time to re-epithelialization was 1.9 months (range, 1.1–3.0 months). The median value of NRS of headache was 8 before surgery and decreased to 0 within 3 months after surgery (*P* < 0.001) (Table 3). Among the enrolled 72 patients, 19 (26.4%) died: 13 (18.1%) due to locoregional recurrence, metastases to distant organs, or both, 2 (2.7%) died from intracranial infection, 3 (4.2%) from nasopharyngeal hemorrhage, and 1 (1.4%) from aspiration pneumonia. Among the 20 patients who were diagnosed with necrosis combined with locoregional recurrence, 12 (60.0%) died: 10 (50.0%) due to locoregional recurrence, metastases to distant organs, or both, one (5.0%) died of intracranial infection and 1 (5.0%) of nasopharyngeal hemorrhage. The 2-year OS rate of the entire cohort was 77.9% (Fig. 3a).

**Table 3:** Distribution of the headache levels measured using the numeric rating scales (NRS) among the patients with postradiation nasopharyngeal necrosis who underwent radical necrectomy followed by reconstruction

Level of NRS	Time point (cases)	
	Before surgery	Within 3 months after surgery
0	8	49
1	0	2
2	1	1
3	3	2
4	1	0
5	4	0
6	7	0
7	8	4
8	13	1
9	10	3
10	17	10

Among the 25 patients whose ICA was found to be exposed in the necrotic lesion, 19 patients had favorable re-epithelialization with no recorded death, and 6 patients showed did not show favorable re-epithelialization, which comprised of 1 died due to tumor progression, 1 from intracranial infection, 1 from aspiration pneumonia, and 3 were still alive at the last follow-up.

### Independent risk factors for the re-epithelialization and survival

From the univariate logistic regression analysis, cycles of radiotherapy (*P* = 0.012), postoperative pathological result (*P* = 0.004), and survival status of the posterior pedicle nasal septum and floor mucoperiosteum flap (*P* < 0.001) were found to be significantly associated with the re-epithelialization of nasopharyngeal defects. Multivariable logistic regression analysis indicated that these three clinicopathological characteristics were also independent predictors of re-epithelialization (Table 1). Next, we further analyzed the predictors of nasopharyngeal defect re-epithelialization in the 52 PRNN patients with negative pathology at the time of surgery and as shown in Table 4, the number of cycles of radiotherapy (*P* = 0.022) and the status of the flap (*P* < 0.001) were significantly associated with the re-epithelialization.

**Table 4:** Predictors of nasopharyngeal defect re-epithelialization of patients with postradiation nasopharyngeal necrosis but with negative pathology for tumor recurrence at the time of surgery

Clinical characteristic	Total [cases (%)]	Status of the re-epithelialization [cases (%)]		Univariate logistic regression analysis	
		Survived	Necrotized	*P*-value	OR (95% CI)
Gender				0.232	2.444 (0.565–10.570)
Female	13 (25.0)	9 (21.4)	4 (40)		
Male	39 (75.0)	33 (78.6)	6 (60)		
Age (years)				0.405	0.551 (0.135–2.241)
< 51	25 (48.1)	19 (45.2)	6 (60)		
≥ 51	27 (51.9)	23 (54.8)	4 (40)		
Cycles of radiotherapy				0.022	5.500 (1.272–23.782)
One	37 (71.2)	33 (78.6)	4 (40)		
Two	15 (28.8)	9 (21.4)	6 (60)		
Necrosis-free interval				0.296	3.194 (0.362–28.180)
Acute necrosis	12 (23.1)	11 (26.2)	1 (10)		
Chronic necrosis	40 (76.9)	31 (73.8)	9 (90)		
Carotid artery exposure				0.978	0.980 (0.240–4.004)
Yes	31 (56.9)	25 (59.5)	6 (60)		
No	21 (40.4)	17 (40.5)	4 (40)		
Osteoradionecrosis				0.173	4.500 (0.517–39.149)
Yes	37 (71.2)	28 (66.7)	9 (90)		
No	15 (28.8)	14 (33.3)	1 (10)		
Status of the flap				< 0.001	180.000 (14.672–2208.254)
Survived	41 (78.8)	40 (95.2)	1 (10)		
Necrotized	11 (21.2)	2 (4.8)	9 (90)		

*OR* odds ratio, *CI* confidence interval

The 2-year OS rate observed in patients with negative postoperative pathology was higher than that in patients with positive postoperative pathology (88.4% vs. 54.2%, *P *< 0.001; Fig. 3b). For patients with surviving flap, their 2-year OS rate was higher than that in patients with a necrotic flap (80.3% vs. 68.8%, *P *= 0.046; Fig. 3c). In addition, postoperative pathological result (hazard ratio [HR], 5.018; 95% CI 1.970–12.782; *P* = 0.001) was an independent prognostic factor for OS in the multivariate Cox model (Table 5). For patients with negative pathology of tumor recurrence in their PRNN lesion at the time of surgery, the 2-year OS rate of those with survived flap was significantly higher than those with necrotic flap (90.1% vs. 81.8%, *P *= 0.043) (Table 6).

**Table 5:** Cox univariate and multivariate analyses of the prognostic factors related to overall survival (OS) for the patients with postradiation nasopharyngeal necrosis

Clinical characteristic	2-year OS rate (%)	Univariate analysis		Multivariate analysis	
		*P* value	HR (95% CI)	*P* value	HR (95% CI)
Gender		0.601	1.295 (0.491–3.412)		
Female	84.0				
Male	76.0				
Age (years)		0.331	1.589 (0.625–4.039)		
< 51	84.7				
≥ 51	72.7				
Cycles of radiotherapy		0.717	1.197 (0.453–3.163)		
One	80.8				
Two	71.9				
Necrosis-free interval		0.617	1.372 (0.397–4.737)		
Acute necrosis	86.3				
Chronic necrosis	75.7				
Carotid artery exposure		0.817	1.122 (0.423–2.975)		
Yes	68.6				
No	82.1				
Osteoradionecrosis		0.607	0.782 (0.308–1.991)		
Yes	81.2				
No	71.4				
Postoperative pathological result		0.001	5.026 (1.974–12.799)	0.001	5.018 (1.970–12.782)
Negative	88.4				
Positive	54.2				
Status of the flap		0.055	2.467 (0.982–6.195)	0.056	2.442 (0.978–6.099)
Survived	80.3				
Necrotized	68.8				

*OS* overall survival, *HR* hazard ratio, *CI* confidence interval

**Table 6:** The prognostic factors for overall survival (OS) for the patients with postradiation nasopharyngeal necrosis with negative pathology at the time of surgery

Clinical characteristic	2-year OS rate (%)	*P*-value
Gender		
Female	100	
Male	84.9	0.613
Age (years)		
< 51	96	
≥ 51	81.6	0.295
Cycles of radiotherapy		
One	86.5	
Two	93.3	1.000
Necrosis-free interval		
Acute necrosis	91.7	
Chronic necrosis	87.7	0.443
Carotid artery exposure		
Yes	75	
No	96.8	0.219
Osteoradionecrosis		
Yes	93.9	
No	73.8	0.374
Status of the NSFF flap		
Survived	90.1	
Necrotized	81.8	0.043
All patients	88.4	

*NSFF* posterior pedicle nasal septum, and floor mucoperiosteum flap

## Discussion

Postradiation nasopharyngeal necrosis has a significant impact on patients’ quality of life and contributes to a high mortality rate [10–12]. Little is known about the best therapeutic management of PRNN. In the present study, we have designed a curative-intent endoscopic surgery comprising of radical endoscopic necrectomy and reconstruction of the nasopharyngeal defect using the nasal septum and floor mucoperiosteum flap and analyzed the treatment outcomes in a cohort of 72 NPC patients. All patients had successful surgical reconstruction of their infected PRNN wounds without any severe postoperative complications or death, of which fifty-one patients (70.8%) achieved successful complete re-epithelialization of their nasopharyngeal defect. The median value of NRS for headache was observed to decrease from a scale of 8 before surgery to scale 0 within 3 months after surgery (*P* < 0.001). The 2-year OS rate of the entire cohort was 77.9%. These results indicated that the proposed surgery could completely remove necrotic tissues, efficiently re-epithelialize nasopharyngeal defects, effectively relieve headache, and increase the OS rate of NPC patients with PRNN lesions, as compared to the results in previously published studies for which the risk of death ranged between 41.8% and 42.9% [9, 11, 12].

Since June 2006, we performed repeated endoscopic debridement of the nasopharyngeal necrotic tissues to obtain fresh wounds, which was helpful to control infections, inhibit necrosis, and relieve symptoms of headache and foul odor [11, 12, 16]. However, these procedures were lengthy and required multiple surgeries. Furthermore, most patients could not achieve complete re-epithelialization of the nasopharyngeal defect. Also, considering that when the ICA is exposed to the PRNN lesions, any aggressive endoscopic debridement is not advocated to prevent the ICA rupture and severe massive bleeding. As such, an efficient technique to remove the necrotic lesion, and to safely and completely re-epithelialize the defect had to be developed as a key step for successful treatment of PRNN lesions.

Endoscopic endonasal approaches have been rapidly developed in recent years. Chen et al. [17, 18]. reported that en-bloc excision of nasopharyngeal tumor lesions could be successfully achieved with endoscopic nasopharyngectomy through endoscopic endonasal approaches for recurrent NPC. Therefore, we thought of applying a similar technique to completely remove the PRNN lesions [17–21]. As such, we designed a novel radical endoscopic necrectomy with curative intent, in which we observed that the 72 patients who underwent this procedure had no severe postoperative complications or death. Moreover, preoperative BOT was used to determine whether the ICA could be safely occluded without cerebral ischemia when the ICA was exposed to the PRNN lesions. Furthermore, endovascular coiling embolization was performed for patients suffering from vascular wall trauma or pseudoaneurysm when the result of BOT was negative. These methods successfully solved the issues regarding intraoperative or postoperative sudden rupture and fatal nasopharyngeal hemorrhage of the ICA and could be additionally helpful to give surgeons enough confidence for the removal of necrotic tissues even they are close to the ICA [14, 15]. Even if the patient had intraoperative or postoperative sudden rupture or even massive nasopharyngeal hemorrhage of the ICA when the BOT result was negative, they could be rescued by endovascular coiling embolization.

In addition, how to effectively and promptly re-epithelialize the nasopharyngeal defect after radical endoscopic debridement is another key issue in treating PRNN. Cornes et al. [22] reported that the use of locoregional pedicle flaps could increase the wound healing rate by 12%–46% as compared with those not using pedicle flaps in the treatment of neck necrosis in patients with head and neck squamous cell carcinoma which was followed by brachytherapy. Hao et al. [23] proposed that healthy vascularized pedicle tissues should be used in reconstruction as this could bring new blood flow and nutrition to assist wound healing and tissue regeneration for the treatment of osteoradionecrosis at the head and neck area. Open salvage surgery following vascularized flap was also reported as an effective treatment for tissue necrosis after irradiation for head and neck cancers [24, 25]. However, until now, there have been no published studies regarding the treatment of PRNN using vascularized free flap in NPC patients.

Vascularized free flap is often used in open surgeries because ample open space is required to insert the micro-vascularized free flap into the nasopharyngeal cavity. However, this type of flap is obviously not suitable for endoscopic endonasal approaches because of the narrow surgical field [13, 26]. We had previously successfully applied nasal septum and floor mucoperiosteum flap to resurface fresh and clean nasopharyngeal defects to promote wound healing in recurrent NPC patients [13]. However, whether the flap would be effective in repairing old and infected necrotic wounds in PRNN remained unknown, until the results of this present study where we demonstrated that the nasal septum and floor mucoperiosteum flap could be useful to repair infected necrotic wounds. Further, we speculated that the survival flap could serve as a barrier to efficiently protect the ICA and as our data demonstrated, the survival flap was a protective factor for the re-epithelialization of the nasopharyngeal defect. We also found that larger number cycles of radiotherapy was an adverse independent predictor of re-epithelialization, and we believe that the reason for this might be because radiotherapy often leads to insufficient local tissue blood flow, oxygen supply, and microcirculation dysfunction; as this was particularly obvious in patients who received two cycles of radiotherapy [8, 23]. Moreover, patients who received two cycles of irradiation developed PRNN faster than those who received one cycle of irradiation (median time to develop PRNN after: one cycle vs. two cycles of irradiation, 8 months vs. 3.5 months, respectively). This suggests that oncologists should be more careful in choosing re-irradiation for recurrent NPC.

During radical endoscopic debridement, we found that some patients presented with recurrent cancer lesion in the deep surgical margins after surgery, despite that these individuals had no signs of recurrence prior to surgery. This finding suggests that superficial histopathological examination may not be sufficient to identify deeply located recurrent lesion, and this phenomenon has not yet been reported in previous studies. Moreover, the postoperative pathologic result was observed to be an independent prognostic factor for OS and a predictor of curing PRNN. For those patients suffering from PRNN combined with recurrent tumor, the treatment strategy would be much more difficult, whereby both the anti-cancer treatment and anti-necrosis treatment would be simultaneously equally important. Therefore, how to differentiate the PRNN from cancerous ulcer is very important. Unfortunately, these lesions are difficult to differentiate solely based on the current imaging techniques, such as MRI and positron emission tomography–computed tomography (PET/CT). In contrast, as demonstrated by our pathological results, we suggest that deep biopsy and/or radical endoscopic surgery might be a better alternative to identify such deeply located recurrence and relieve associated headaches. Based on these findings, we recommend radical endoscopic surgery for patients with PRNN, whether combined with or without deeply recurrent tumor.

Despite the promising results of this present study, there are several potential limitations worth mentioning. First, the data of this study were retrospectively analyzed, and prospective studies with a larger cohort are required to validate these results. Second, the number of patients was limited, and all of them were treated at a single cancer center. Third, the median follow-up time was relatively short (20 months), and we expect future studies to have a longer follow-up time.

## Conclusions

Curative-intent endoscopic necrectomy followed by reconstruction using the posterior pedicle nasal septum and floor mucoperiosteum flap is a novel, safe, and effective treatment of PRNN in patients with NPC. However, it should be further validated with a long follow-up period.

## Abbreviations

PRNN: postradiation nasopharyngeal necrosis
NPC: nasopharyngeal carcinoma
OS: overall survival
OR: odds ratio
CI: confidence interval
HR: hazard ratio
ICA: internal carotid artery
BOT: balloon occlusion test
MRI: magnetic resonance imaging
NRS: numeric rating scales
PET/CT: positron emission tomography–computed tomography
ET: eustachian tube
NSFF: posterior pedicle nasal septum, and floor mucoperiosteum flap
